# Supplementary material for: WordCommentsAnalyzer: A windows software tool for qualitative research
Source: F1000Res. 2018 Sep 4;7:536. Originally published 2018 May 3. [Version 2] doi: 10.12688/f1000research.14819.2 (PMC6137411; doi:10.12688/f1000research.14819.2)
Supplement: Supplementary file 3 [file f1000research-7-17614-s0002.tgz › b2a79496-4ada-47c4-9682-a99ce867836d.zip!/Eight Iranian Health Journals/Journal of Health System Research _3.docx]

© Journal of Health System Research . This work is licensed under CC BY-NC 4.0 (https://creativecommons.org/licenses/by-nc/4.0/).

Volume & Issue: دوره 13، شماره 3، 1396

Title: مکمل یاری تغذیه‌ای در بیماران مزمن کلیوی: مروری بر شواهد موجود

http://hsr.mui.ac.ir/index.php/jhsr/article/view/3156

مشکلاتی از جمله اختلالات هورمونی، التهاب و محدودیت‌های رژیم‌های غذایی، می‌تواند منجر به سوء تغذیه در بیماران مبتلا به بیماری مزمن کلیه به ویژه بیماران تحت دیالیز شود. بهبود وضعیت تغذیه این بیماران با مداخلات رژیمی و یا غیر رژیمی، گامی حیاتی در کاهش میزان عوارض و مرگ و میرهای ناشی از این بیماری می‌باشد. اختلاف نظرهای بسیاری در مورد استفاده از مکمل‌های ویتامینی در بیماری‌های کلیوی وجود دارد و در این زمینه توصیه‌های مبتنی بر شواهد علمی، بسیار اندک است. با توجه به موارد مطرح ‌شده و افزایش مصرف روزافزون مکمل‌های غذایی در جهان، مطالعه‌ مروری حاضر با هدف جمع‌آوری و خلاصه‌سازی شواهد علمی موجود در مورد مکمل یاری‌های تغذیه‌ای در بیماران مزمن کلیوی انجام گردید.

Title: رابطه نمره شاخص استرین گرمایی و ضربان قلب با اضافه وزن تحت شرایط گرم آزمایشگاهی

http://hsr.mui.ac.ir/index.php/jhsr/article/view/2391

مقدمه: يكي از شاخص‌هاي ارزیابی میزان استرین گرمایی، نمره شاخص استرين گرمایي (Heat strain scoring index یا HSSI) می‌باشد. پژوهش حاضر با هدف بررسی ارتباط نمره HSSI و ضربان قلب در افراد دارای اضافه وزن در دو شدت فعالیت سبک و متوسط در شرایط گرم آزمایشگاهی انجام شد. روش‌ها: 35 فرد دارای اضافه وزن و 35 فرد دارای وزن طبیعی در این مطالعه تجربی شرکت کردند. پس از استراحت، شخص به مدت 60 دقیقه یک بار با سرعت 8/2 کیلومتر در ساعت بر روی تردمیل در مواجهه با شرایط خیلی گرم (دمای 37 درجه سانتی‌گراد) و بار دیگر با سرعت 8/4 کیلومتر در ساعت بر روی تردمیل در مواجهه با شرایط گرم (دمای 32 درجه سانتی‌گراد) فعالیت نمود. ضربان قلب هر 2 دقیقه اندازه‌گیری گردید. پرسش‌نامه HSSI در سه مرحله زمانی زمان استراحت، پس از پایان 25 دقیقه فعالیت اول و پس از پایان 25 دقیقه فعالیت دوم تکمیل شد. یافته‌ها: نتایج ضریب همبستگی Pearson نشان داد که در شرایط گرم، بین ضربان قلب فعالیت اول و نمره HSSI (001/0 > P، 50/0 = r) و ضربان قلب فعالیت دوم و نمره HSSI (001/0 > P، 64/0 = r) رابطه خطی مستقیمی وجود داشت. همچنین، ارتباط خطی مستقیمی بین ضربان قلب فعالیت اول و نمره HSSI (001/0 > P، 47/0 = r) و ضربان قلب فعالیت دوم و HSSI (001/0 > P، 64/0 = r) در شرایط خیلی گرم مشاهده شد. نتیجه‌گیری: میانگین ضربان قلب و شاخص HSSI در افراد داراي اضافه وزن در مقايسه با افراد با وزن طبیعی، بالاتر بود و ضربان قلب همبستگی قابل توجهی را با این شاخص نشان داد.

Title: اثر روغن‌های گیاهی مختلف بر خواص حسی و کیفی کنسرو ماهی تون

http://hsr.mui.ac.ir/index.php/jhsr/article/view/2990

مقدمه: کنسرو کردن، یکی از روش‌های نگهداری ماهی تون می‌باشد. از آن‌جایی که روغن ماهی به واکنش اکسیداسیون حساس است و روغن در مرحله پخت مقدماتی از بافت ماهی جدا می‌شود؛ طبق استاندارد، 14 تا 18 درصد روغن گیاهی به کنسرو ماهی تون افزوده می‌شود. دمای بالا و زمان طولانی فرایند کنسروسازی، موجب آسیب به روغن موجود در کنسرو مواد غذایی به ویژه ماهی می‌گردد. بنابراین، با استفاده از روغن‌های گیاهی مقاوم به اکسیداسیون، می‌توان آسیب حرارتی روغن را تا میزان زیادی کاهش داد. روش‌ها: روغن‌های گیاهی شامل روغن زیتون، کنجد، ذرت و بادام زمینی به میزان 14، 16 و 18 درصد به قوطی حاوی ماهی افزوده شد و پس از فرایند کنسروسازی، خصوصیات کیفی شامل ترکیب اسیدهای چرب، اندیس پراکسید، شاخص تیوباربیتوریک اسید و بافت و ویژگی‌های حسی با کنسروهای رایج در بازار (روغن سویا با میزان 18 درصد) مقایسه گردید. یافته‌ها: فرایند کنسروسازی باعث کاهش دو اسید چرب ایکوزاپنتانوئیک اسید (Eicosapentaenoic acid یا EPA) و دکوزاهگزانوئیک اسید (Docosahexaenoic acid یا DHA) ماهی شد. بالاترین پایداری اکسیداسیونی به کنسرو ماهی تولید شده با روغن کنجد غلظت 18 درصد اختصاص داشت. همچنین، بالاترین میزان پذیرش از دیدگاه ارزیاب‌های حسی، غلظت 18 درصد روغن کنجد و زیتون در فرآورده نهایی بود.نتیجه‌گیری: از آن‌جایی که کنسرو ماهی تون حاوی 18 درصد روغن کنجد، بهترین کیفیت و ارزش تغذیه‌ای را داشت، جایگزینی روغن سویا با روغن کنجد در تولید کنسرو ماهی تون پیشنهاد می‌شود.

Title: بررسی تغییرات جمعیت میکروبی خاک در فرایند گیاه پالایی خاک آلوده به نفت خام

http://hsr.mui.ac.ir/index.php/jhsr/article/view/2955

مقدمه: گیاه پالایی روش ارزان و مؤثری جهت تصفیه خاک آلوده به نفت خام می‌باشد. به منظور اجرای هرچه بهتر این فرایند، آگاهی از روند تغییرات جمعیت میکروبی ضروری است. هدف از انجام پژوهش حاضر، بررسی تغییرات جمعیت میکروبی در خاک ریزوسفری و شاهد بود. روش‌ها: خاک مورد مطالعه به پنج قسمت تقسیم و با غلظت‌های 43/0، 86/0، 9/1، 13/4 و 27/8 درصد وزنی- وزنی نفت خام، آلوده گردید. سپس تیمارهایی از آن تهیه شد و تغییرات جمعیت میکروبی خاک به مدت شش ماه مورد بررسی قرار گرفت. جمعیت میکروبی و غلظت اولیه نفت خام به ترتیب به روش شمارش بشقابی هتروتروفیک (Heterotrophic plate count یا HPC) و به وسیله دستگاه کروماتوگرافی گازي (Gas Chromatography یا GC) سنجش شد. یافته‌‌ها: میانگین جمعیت میکروبی در تیمارهای کشت شده با گیاه [log10 55/7 واحد تشکیل کلونی (Colony-forming unit یا CFU) بر گرم] و تیمارهای حاوی مواد مغذی (log10 79/7 واحد تشکیل کلونی بر گرم) به طور معنی‌داری بیشتر از تیمارهای بدون گیاه (log10 629/6 واحد تشکیل کلونی بر گرم) و بدون مواد مغذی (log10 97/6 واحد تشکیل کلونی بر گرم) بود (05/0 > P). حداکثر جمعیت میکروبی نیز درتیمارهای آلوده به غلظت 86/0 (log10 372/8 واحد تشکیل کلونی بر گرم) به دست آمد. نتیجه‌‌گیری: بر اساس نتایج حاصل شده، وجود گیاه و مواد مغذی به دلیل فراهم نمودن ریزمغذی برای باکتری‌ها، می‌تواند جمعیت میکروبی خاک و در نتیجه، کارایی گیاه پالایی را افزایش دهد.

Title: ارزيابي ريسك سلامتی آرسنیک در منابع آب آشاميدني و آبیاری و برنج منطقه میداوود در استان خوزستان

http://hsr.mui.ac.ir/index.php/jhsr/article/view/2876

مقدمه: آلودگی منابع آب به فلزات سنگین و ورود به چرخه زیستی می‌تواند اثرات مخرب زیست‌محیطی را به دنبال داشته باشد. آرسنیک در آب آشامیدنی از جمله عناصری است که غلظت بالای آن پیامدهای نابهنجاری را برای سلامتی بدن به دنبال دارد. بنابراین، مطالعه حاضر با هدف تعيين ريسك سلامتی آرسنیک در منابع آب شرب، آبیاری و برنج منطقه میداوود در استان خوزستان صورت گرفت. روش‌ها: از چاه‌های آب شرب، آبیاری و همچنین، از دانه‌های برنج موجود در مزارع منطقه نیز نمونه‌برداری به عمل آمد. پس از آماده‌سازی نمونه، سنجش غلظت آرسنیک با دستگاه (ICP-MS یا Inductively coupled plasma mass spectrometry) انجام شد. یافته‌ها: میانگین کل غلظت آرسنیک در نمونه‌های آب شرب، آبیاری و برنج منطقه میداوود به ترتیب 8، 75/12 (میلی‌گرم بر لیتر) و 079/0 (میلی‌گرم بر کیلوگرم) حاصل شد. جذب روزانه مزمن (CDI یا Chronic daily intake) آرسنیک از طریق مصرف آب و برنج، برای گروه کودکان و زنان و مردان محاسبه گردید که کمتر از حد قابل تحمل دریافت روزانه FAO (Food and agriculture organization) بود. همچنین، نتایج سهم خطر در هر سه گروه (1 <) به دست آمد که در محدوده نگران کننده و غیر ایمن است. نتیجه‌گیری: به طور کلی، سهم خطر (1 <) برای ساکنان منطقه به ویژه گروه کودکان به جهت آشامیدن آب و مصرف برنج در محدوده نگران کننده و غیر ایمن قرار دارد. توجه به این نکته که آب شرب و آبیاری مزارع منطقه میداوود از چاه تأمین می‌شود و تغییرات دبی و سطح ایستابی چاه بر میزان غلظت آرسنیک اثرگذار است، کنترل منابع آب در این منطقه ضرورت دارد.

Title: تأثیر مداخله آموزشی مبتنی بر تئوری شناختي اجتماعي بر رفتار دست شستن دانش‌آموزان دبستانی

http://hsr.mui.ac.ir/index.php/jhsr/article/view/2978

مقدمه: یکی از مناسب‌ترین و مقرون به صرفه‌ترین راه‌ها جهت ممانعت از گسترش عفونت، شستن دست‌ها می‌باشد. مدرسه یکی از محیط‌های مناسب برای مداخله با هدف ارتقای رفتارهای بهداشتی محسوب می‌شود. انجام آموزش‌های مؤثر در این زمینه به کودکان، می‌تواند منجر به کاهش بیماری‌های عفونی تنفسی و گوارشی، کاهش هزینه‌های بهداشتی و درمانی و کاهش مقاومت آنتی‌بیوتیکی گردد. روش‌ها: تحقيق حاضر از نوع نیمه تجربی بود که با دو گروه آزمون و شاهد بر روی 68 دانش‌آموز پایه سوم دبستان انجام شد. متغیرهای دموگرافیک و متغیرهای مبتنی بر سازه‌های تئوری شناختي اجتماعي شامل الگوسازي، تنظيم هدف، غلبه بر موانع، محیط و خودكارامدي مورد بررسي قرار گرفت. داده‌های جمع‌آوری شده با استفاده از آزمون‌های توصیفی و استنباطی در نرم‌افزار SPSS تجزیه و تحلیل گردید. یافته‌ها: افزايش معنی‌داری در ميانگين تعداد دفعات شستن دست‌ها در طول روز، بعد از انجام مداخله در گروه آزمون نسبت به قبل از مداخله وجود داشت (041/0 = P). همچنین، افزایش معنی‌داری در تعداد دفعات دست شستن گروه آزمون نسبت به گروه شاهد مشاهده گردید (046/0 = P). تأثیر مداخله آموزشی بر عوامل شناختی اجتماعی شامل افزایش آگاهی (048/0 = P) و حمایت اجتماعی (008/0 = P) در پایان مطالعه در گروه آزمون نسبت به گروه شاهد معنی‌دار بود. میانگین نمره آگاهی دانش‌آموزان در گروه آزمون پس از انجام مداخله نسبت به قبل از آن، افزایش معنی‌داری را نشان داد (034/0 = P). نتیجه‌گیري: آموزش و حمایت اجتماعي در چهارچوب تئوری شناختی اجتماعی، می‌تواند مداخله مناسبی در خصوص رفتار دست شستن در دانش‌آموزان باشد.

Title: بررسی آثار نامتقارن نوسانات قیمت نفت بر مخارج دولت در بخش بهداشت و سلامت در ایران

http://hsr.mui.ac.ir/index.php/jhsr/article/view/3058

مقدمه: بررسی رفتار دولت در نحوه تخصیص منابع مالی به بخش سلامت به هنگام تغییرات مثبت و منفی در قیمت نفت از بعد سیاستگذاری حوزه بهداشت و سلامت دارای اهمیت فراوانی است. از این‌رو، هدف از انجام مطالعه حاضر، بررسی اثر نامتقارن نوسانات قیمت نفت بر مخارج دولت در بخش سلامت طی دوره 1392-1375 در ایران بود. روش‌ها: برای بررسی اثر نامتقارن نوسانات قیمت نفت بر مخارج دولت در بخش بهداشت و سلامت ابتدا تلاش شد با استفاده از یک مدل خودرگرسیو واریانس ناهمسان شرطی تعمیم یافته (GARCH یا Generalized auto-regressive conditional heteroscedasticity) نوسانات مثبت و منفی قیمت نفت محاسبه و سپس، با استفاده از رویکرد اقتصادسنجی Bayesian و توابع واکنش به ضربه (Response function)، تأثیر هر یک از نوسانات مثبت و منفی بر مخارج دولت در بخش سلامت مورد بررسی قرار گیرد. یافته‌ها: هم در زمان افزایش قیمت نفت و هم زمان کاهش قیمت، دولت مخارج خود در بخش سلامت را افزایش داده و این مسأله نشان دهنده اهمیت بخش بهداشت و سلامت به عنوان یکی از شاخص‌های مهم توسعه انسانی در سیاستگذاری‌های مربوط به تخصیص منابع در دوره مذکور از سوی دولت است. نتیجه‌گیری: نوسانات قیمت نفت به عنوان یکی از عوامل ایجاد بی‌ثباتی در تأمین منابع درآمدی دولت در بودجه به شمار می‌آید. از این‌رو، تأمین مخارج بخش بهداشت و سلامت به عنوان یکی از شاخص‌های اثرگذار بر توسعه انسانی از سوی دولت ممکن است با بی‌ثباتی‌هایی روبه‌رو شود. به همین منظور، مطالعه حاضر نشان می‌دهد که دولت در دوره مذکور تلاش نموده است تا تخصیص منابع به این بخش دچار بی‌ثباتی نشود.

Title: بررسی کیفی روغن‌های مصرفی و باقی‌مانده در محصولات غذایی تهیه شده در اغذیه فروشی‌ها و قنادی‌های شهرستان‌های شاهین‌شهر و میمه

http://hsr.mui.ac.ir/index.php/jhsr/article/view/3006

مقدمه: حرارت دادن زیاد روغن‌های خوراکی در حضور هوا، سبب تغییرات اکسیداتیو و تولید هیدروپراکسیدها و ترکیبات فرار خطرناک در آن‌ها می‌گردد. مطالعه حاضر با هدف تعیین میزان عدد پراکسید، رانسیدیته و مقایسه این دو شاخص در روغن‌های مصرفی و باقی‌مانده در قنادی‌ها و اغذیه فروشی‌های شهرستان‌های شاهین‌شهر و میمه در سال 1391 صورت گرفت. روش‌ها: در این مطالعه، 103 نمونه روغن از کارگاه‌های قنادی و اغذیه فروشی‌ها به صورت تصادفی جمع‌آوری شد. نمونه‌گیری وآزمایش پراکسید به ترتیب طبق روش‌های شماره 493 و 4179 استاندارد ایران و آزمایش رانسیدیته نیز طبق روش  Kereisانجام گرفت. جهت بررسی صحت نتایج، تعدادی از نمونه‌ها به طور هم‌زمان به سه آزمایشگاه دانشکده بهداشت، خصوصی ابن‌سینا و غذا و داروی اصفهان ارسال گردید. یافته‌ها: 100 درصد روغن‌های خام و 3/88 درصد روغن‌های پخته شده و استخراجی، از نظر عدد پراکسید مجاز بودند. از نظر رانسیدیته نیز 100 درصد نمونه روغن‌های خام سالم بودند؛ در حالی که 100 درصد نمونه روغن‌های پخته شده و 0/85 درصد روغن‌های استخراج شده فاسد بودند. همچنین، 8/16 و 0/75 درصد نمونه‌های گرفته شده از واحدهای با معیار بهداشتی و بهسازی پراکسید بالاتر از حد مجاز داشتند. نتایج به دست آمده از آزمایشگاه دانشکده بهداشت و ابن‌سینا، تطابق کاملی را با هم نشان داد. نتیجه‌گیری: انجام آزمایش پراکسید به تنهایی جهت تعیین کیفیت روغن‌ها کافی نیست و باید از آزمایش‌های دیگری مانند رانسیدیته نیز استفاده نمود. همچنین، وضعیت بهداشتی واحدهای تولیدی، نقش بسزایی در بهبود این شاخص‌ها دارند.

Title: بررسی آلودگی میکروبی و شیمیایی آب آشامیدنی عرضه شده در اتوبوس‌های مسافربری ترمینال صفه اصفهان: یک مطالعه موردی

http://hsr.mui.ac.ir/index.php/jhsr/article/view/2999

مقدمه: امروزه اتوبوس‌های بین شهری نقش مهمی در انتقال مسافران دارند که در صورت آلوده بودن آب شرب آن‌ها، سلامتی مسافران به خطر می‌افتد. مطالعه حاضر با هدف بررسی کیفیت میکروبی و شیمیایی آب آشامیدنی عرضه شده در اتوبوس‌های بین شهری ترمینال صفه اصفهان انجام شد. روش‌ها: در این مطالعه، 60 نمونه از آب آشامیدنی عرضه شده در اتوبوس‌ها به صورت تصادفی جمع‌آوری گردید و آلودگی میکروبی آن‌ها با آزمایش‌های Most probable number (MPN) به روش تخمیر 9 لوله‌ای مورد بررسی قرار گرفت. همچنین، شاخص‌های شیمیایی شامل هدایت الکتریکی (Electrical conductivity یا EC)، کل جامدات محلول (Total dissolved solids یا TDS)، سختی کل، كلر باقی‌مانده، كدورت، pH و دما مطابق روش‌های استاندارد سنجیده شد. داده‌ها در نرم‌افزار SPSS تجزیه و تحلیل گردید. یافته‌ها: در 20 درصد از نمونه‌ها، کلیفرم کل و در 7 درصد آن‌ها کلیفرم مدفوعی مثبت بود و تمام آلودگی‌ها به آبسردکن‌ها اختصاص داشت. میزان EC در 35 درصد نمونه‌ها بیشتر از 500 میکروموس بر مترمربع، کدورت در 6/11 درصد نمونه‌ها بالاتر از استاندارد 1 واحد اندازه‌گیری کدورت (Nephelometric turbidity unit یا NTU) و کلر باقی‌مانده نیز در 3/98 درصد از نمونه‌ها صفر به دست آمد. نتیجه‌گیری: بیشترین موارد آلودگی به کلیفرم مدفوعی مربوط به آبسردکن اتوبوس‌ها بود و آلودگی در هیچ‌کدام از آب‌های بطری شده مشاهده نگردید. بنابراین، جمع‌آوری آبسردکن و عرضه آب آشامیدنی بطری شده در اتوبوس‌های بین شهری، می‌تواند سهم بسزایی در کاهش بیماری‌های گوارشی داشته باشد.

Title: فراوانی اکراتوکسین A در آردهای مصرفی نان سنگک و اثر فرایندهای تخمیر و پخت بر آن

http://hsr.mui.ac.ir/index.php/jhsr/article/view/3117

مقدمه: اکراتوکسین A نوعی سم قارچی با خطرات اثبات شده‌ای برای سلامتی انسان است و گندم و مواد غذایی مشتق از آن می‌توانند ناقل حدودی از این سم به رژیم غذایی انسان باشند. هدف از انجام مطالعه حاضر، بررسی میزان فراوانی اکراتوکسین A در نمونه‌های آرد نان سنگک و بررسی اثر عملیات نانوایی بر غلظت موجود در آلوده‌ترین نمونه‌های آرد بود. روش‌ها: غلظت اکراتوکسین در 30 نمونه آرد سنگک با روش Enzyme-linked immunosorbent assay (ELISA) رقابتی و با استفاده از کیت تجارتی اندازه‌گیری شد. آلوده‌ترین نمونه‌ها تحت فرایند نانوایی شامل تخمیر با مخمر نانوایی ساکارومایسیس سروزیه و تخمیر با مخمر همراه با لاکتوباسیلوس پلانتارم A7 (Lactobacillus plantarum A7 یا L. plantarum A7) قرار گرفتند و غلظت اکراتوکسین در طول فرایند تهیه نان سنگک اندازه‌گیری گردید. یافته‌ها: آلودگی به اکراتوکسین A در کلیه نمونه‌های آرد مورد آزمایش مشاهده شد. محدوده غلظت و میانگین اکراتوکسین به ترتیب 723/1-(Limit of Determination) LOD و 025/1 نانوگرم بر گرم به دست آمد. هر دو فرایند تخمیر و پخت، به طور معنی‌داری سم اکراتوکسین را کاهش داد (05/0 > P) و غلظت هیچ یک از نمونه‌ها بیش از حداکثر مجاز تعیین شده توسط اتحادیه اروپا (5 نانوگرم بر گرم) نبود. در فرایند تخمیر با مخمر ساکارومایسس سرویزیه و مخلوط مخمر و لاکتوباسیلوس پلانتاروم، به ترتیب کاهشی معادل 7 و 15 درصد مشاهده شد. فرایند پخت نیز میزان اکراتوکسین را 24 درصد کاهش داد. نتیجه‌گیری: نمونه‌های آرد و نان سنگک موجود در بازار از نظر آلودگی به اکراتوکسین A، باید آلودگی پایین‌تر از حد مجاز داشته باشند. فرایند پخت، تخمیر با مخمر و افزودن کشت لاکتیکی به محیط تخمیر، اکراتوکسین را به طور معنی‌داری کاهش می‌دهد.

Title: بررسی کیفیت تشخیص میکروسکوپیست‌های شاغل در آزمایشگاه تشخیص سل ریوی در استان اصفهان در سال 1394

http://hsr.mui.ac.ir/index.php/jhsr/article/view/2953

مقدمه: بیماری سل از جمله بیماری‌های عفونی مهم است و تشخیص، درمان و کنترل آن در جامعه بسیار مهم و حیاتی می‌باشد. از طرف دیگر، وجود افراد با تجربه در تشخیص بیماری از اهمیت بسیاری برخوردار است. از این‌رو، در مطالعه حاضر کیفیت کار تشخیص میکروسکوپیست‌های شاغل در آزمایشگاه‌های محیطی استان اصفهان در سال 1394 مورد بررسی قرار گرفت. روش‌ها: در مطالعه توصیفی- تحلیلی که با انجام یک آزمون عملی در بین 27 نفر شاغل در 20 آزمایشگاه محیطی بخش تشخیص اسمیر مستقیم سل ریوی استان اصفهان انجام شد، از طریق ارسال 5 لام اسمیر مستقیم خلط (با درجات متفاوتی از وجود باسیل سل و یا عدم وجود) همراه با یک فهرست وارسی و سپس، جمع‌آوری جواب‌های آزمون پس از مهلت چند روزه صورت گرفت. یافته‌ها: بین کیفیت تشخیص با سن، جنس، وضعیت استخدام شاغلان، اختصاصی بودن میکروسکوپ مورد استفاده و عمر میکروسکوپ و همچنین، فعالیت‌های میکروسکوپیست در بخش‌های دیگر آزمایشگاه رابطه معنی‌داری وجود نداشت، اما میزان تحصیلات دانشگاهی، سابقه فعالیت شاغلان در بخش سل و مدت زمانی که صرف دیدن هر لام اسمیر مستقیم شد، بر کیفیت تشخیص اثرگذار بود. در بین افراد مورد پژوهش به طور تقریبی 75 درصد افراد در آزمون نتیجه مطلوب کسب کردند. نتیجه‌گیری: از آن‌جایی که مشاهده اسمیر مستقیم سل هنوز یکی از در دسترس‌ترین و سریع‌ترین روش تشخیص سل ریوی است، پژوهش فوق به این نتیجه دست یافت که کیفیت تشخیص این افراد باید حداقل به صورت سالانه توسط آزمایشگاه رفرانس سل استان مورد ارزیابی قرار گیرد.

Title: پهنه‌بندی غلظت نیترات آبخوان میمه با استفاده از سيستم اطلاعات جغرافيايي

http://hsr.mui.ac.ir/index.php/jhsr/article/view/3037

مقدمه: امروزه افزايش غلظت تركيبات نيتريت و نيترات در منابع آب زيرزميني، باعث ايجاد نگراني‌هایي در محیط‌های کشاورزی و صنعتی شده است. نيترات به عنوان يكي از شاخص‌هاي شيميايي آلودگي آب، از ديرباز مورد توجه بوده است. هدف از انجام مطالعه حاضر، تعيين مقادير نيترات در آبخوان ميمه در استان اصفهان بود. روش‌‌ها: از 10 حلقه چاه بر اساس اصول نمونه‌برداري استاندارد، نمونه گرفته شد و نمونه‌ها جهت آنالیز مقدار نیترات به آزمایشگاه آب منتقل گردید. اندازه‌گيري مقدار نيترات به روش اسپكتروفتومتري صورت گرفت. سپس پهنه‌بندی غلظت نیترات با استفاده از نرم‌افزار ArcGIS و مدل‌های میان‌یابی ترسیم شد. یافته‌ها: میزان هدايت الكتريكي (Electrical conductivity یا EC) چاه‌هاي انتخابي بین 823 تا 4170 میکروزیمنس بر سانتی‌متر و ميانگين غلظت نيترات 77/19 ± 64/30 میلی‌گرم در لیتر به دست آمد. حداقل و حداکثر غلظت نیترات به ترتیب 51/8 و 43/59 میلی‌گرم در لیتر بود. انحراف معیار در 68 درصد داده‌ها مشاهده گردید که پراکندگی نزدیک به میانگین را نشان داد. نتیجه‌گیری: میزان EC نمونه‌های آب از دامنه ارتفاعات و نواحي تغذيه دشت در شمال خاوري و باختري به سمت مركز دشت افزايش مي‌يابد. همچنین، بالاترين غلظت نيترات به قسمت جنوبی آبخوان اختصاص داشت که به احتمال زیاد ناشي از بالا بودن سطح آب زيرزميني و فعاليت‌های کشاورزي در منطقه می‌باشد. اگرچه ميانگين غلظت نيترات در نمونه‌ها از 50 میلی‌گرم در لیتر كمتر است، اما با اين وجود حفاظت از منابع آب زيرزميني در برابر ورود آلاينده‌ها ضروري به نظر مي‌رسد. كليد واژه‌ها: نیترات، آبخوان ميمه ، سيستم اطلاعات جغرافيايي

Title: تأثیر امواج اولتراسونیک بر میزان درصد جامدات لجن بیولوژیکی در فرایند آبگیری لجن مازاد

http://hsr.mui.ac.ir/index.php/jhsr/article/view/3043

مقدمه: امروزه آبگیری لجن، یکی از مشکل‌ترین فرایندها در تصفیه‌خانه‌های فاضلاب و مسأله مهمی جهت افزایش میزان تصفیه و کاهش آلودگی محیط‌ زیست می‌باشد. هدف از انجام تحقیق حاضر، تعیین اثر سیستم انتشار امواج اولتراسونیک جهت بهبود آبگیری لجن، افزایش میزان جامدات در کیک لجن و تعیین محدوده بهینه شاخص‌های راهبری در استفاده از روش‌ اولتراسونیک بود. روش‌ها: این مطالعه به روش تجربی- آزمایشگاهی و به صورت ناپیوسته انجام شد و در آن تأثیر متغیرهایی مانند چگالی اولتراسوند (بر حسب وات بر میلی‌لیتر)، زمان (بر حسب دقیقه) و میزان لجن قرار گرفته در معرض سونیفیکاسیون (بر حسب درصد)، بر میزان جامدات کیک و آبگیری لجن مورد بررسی قرار گرفت. میزان جامدات در نمونه‌های لجن قبل از آبگیری، 9/0 ± 3/20 درصد به دست آمد. داده‌ها در نرم‌افزار SPSS تجزیه و تحلیل گردید. یافته‌ها: میزان بهینه زمان سونیفیکاسیون، 5 دقیقه و چگالی اولتراسوند 1 وات بر میلی‌لیتر مشخص شد. همچنین، میزان لجن قرار گرفته در معرض سونیفیکاسیون، 15 درصد و فرکانس حاصل شده، 20 کیلوهرتز بود. میزان جامدات کیک لجن در این شرایط، 4/26 درصد به دست آمد که معادل 30 درصد بهبود در آبگیری لجن بود. نتیجه‌گیری: امواج اولتراسونیک و میکرو و نانوحباب‌های کاویتاسیون، قابلیت افزایش میزان جامدات کیک لجن را دارند و می‌توانند سبب بهبود فرایند آبگیری و افزایش راندمان تصفیه لجن شوند.

Title: بررسی تأثیر استفاده از سایلنسر جذبی محور مرکزی در کاهش صدای کانال ورودی هوای فن دمنده سانتریفوژ

http://hsr.mui.ac.ir/index.php/jhsr/article/view/2926

مقدمه: فن‌های سانتریفوژ، از جمله فن‌های پرمصرف در واحدهای صنعتی به منظور انتقال حجم زیادی از هوا به تجهیزات صنعتی مانند بویلرها، بلوایر و... می‌باشند که از مهم‌ترین منابع صدا در صنعت به شمار می‌روند. مطالعه حاضر با هدف بررسی تأثیر استفاده از سایلنسر جذبی در کاهش صدای فرکانس پایین فن سانتریفوژ ایرانی انجام شد. روش‌ها: فن مورد بررسی دارای قطر 55 سانتی‌متر با مشخصات فنی شامل سرعت 2980 دور و دبی هوای عبوری 3000 مترمکعب بر ساعت بود. جنس دیواره سایلنسر از آهن گالوانیزه با ضخامت 3 میلی‌متر تشکیل شد. سایلنسر با محور مرکزی به قطر 5 سانتی‌متر و طول 100 سانتی‌متر طراحی گردید و داخل سایلنسر و اطراف محور مرکزی آن با پشم شیشه به عنوان جاذب صوت پوشانده شد. یافته‌ها: حداکثر کاهش فشار صوت در فرکانس‌های پایین با استفاده از سایلنسر جذبی طراحی شده، 12 دسی‌بل به دست آمد. با افزایش قطر و چگالی جاذب، نتایج بهتری در میزان کاهش تراز فشار صوت مشاهده شد. نتیجه‌گیری: با توجه به نتایج به دست آمده در خصوص کاهش صدای فن‌های سانتریفوژ، می‌توان از سایلنسرهای جذبی محور مرکزی با مشخصات جاذب پشم شیشه به ضخامت 10 سانتی‌متر و دانسیته 40 کیلوگرم بر مترمکعب، جهت کاهش فشار صوت در فرکانس‌های مختلف به عنوان وسیله مؤثری استفاده نمود.

Title: بررسی ارتباط بار ذهنی کار و رضایت شغلی در تکنسین‌های مراکز فوریت‌های پزشکی شهر اصفهان در سال 1394

http://hsr.mui.ac.ir/index.php/jhsr/article/view/3026

مقدمه: پرسنل فوریت‌های پزشکی هنگام انتقال بیمار از محل حادثه تا مراکز درمانی باید دارای سرعت عمل و تصمیم‌گیری حیاتی، نظارت بر هوشیاری و علایم حیاتی وی باشند. مطالعه حاضر با هدف بررسی میزان بار ذهنی کار، ارتباط و درجه تأثیر آن بر فاکتور رضایت شغلی در تکنسین‌های مراکز فوریت‌های پزشکی انجام شد. روش‌ها: مطالعه توصیفی- تحلیلی حاضر از نوع مقطعی بود که بر روی 82 نفر از پرسنل فوریت‌های پزشکی شهر اصفهان صورت گرفت. برای جمع‌آوری داده‌ها از نرم‌افزار NASA-TLX (The NASA task load index) به منظور بررسی بار ذهنی کار و پرسش‌نامه رضایت شغلی Visoki و Chrome برای سنجش رضایت شغلی استفاده گردید. آنالیز داده‌ها باآزمون t زوجی، ضریب همبستگی Pearson و نرم‌افزار SPSS انجام پذیرفت. یافته‌ها:کمترین میانگین نمره رضایت شغلی مربوط به پرداخت (9/1) بود. میانگین نمره بار ذهنی کار افراد در عملیات ساده (76/57) و پیچیده (87/82) به دست آمد. نمره رضایت شغلی کلی فقط با نمره ارزیابی عملکرد در عملیات ساده و پیچیده ارتباط مستقیمی داشت. ارتباط نمره کلی بار ذهنی با نمره رضایت شغلی در بعد کار مستقیم بود، اما در بقیه حیطه‌ها معنی‌دار نبود (05/0 < P). در ضمن، در آزمون t زوجی، میانگین نمره کلی بار ذهنی کار و تمامی ابعاد آن در عملیات پیچیده به طور معنی‌داری بیشتر از عملیات ساده بود (05/0 > P). نتیجه‌گیری: بار ذهنی کار در عملیات پیچیده می‌تواند به عنوان عاملی برای تعیین میزان سختی کار و ساعت کاری مناسب برای تکنسین‌های فوریت پزشکی استفاده شود. با توجه به بالا بودن نارضایتی افراد در بخش حقوق، مزایا و ارتقا، شاید بتوان با بهبود شرایط کاری پرسنل از طریق بازنگری میزان پرداخت حقوق و مزایا، رضایت شغلی این قشر مهم و حساس جامعه را افزایش داد.

Title: مکان‌یابی محل دفن پسماند شهری با استفاده از روش وزن‌دهی سلسله مراتبی معکوس

http://hsr.mui.ac.ir/index.php/jhsr/article/view/2958

مقدمه: شهر سنندج با جمعیتی معادل 370 هزار نفر، روزانه 330 تن زباله تولید می‌کند. محل کنونی دفن زباله این شهر علاوه بر تکمیل ظرفیت آن، اثرات نامطلوب محیط زیستی فراوانی را نیز بر محیط اطراف داشته است. بنابراین، مطالعه حاضر با هدف ارزیابی میزان مطلوبیت اراضی شهرستان سنندج جهت دفن پسماندهای جامد انجام شد. مکان‌یابی محل دفن زباله، یک آنالیز تصمیم‌گیری چند معیاره مکانی (Spatial Multi Criteria Decision Analysis یا SMCDA) است که از سیستم اطلاعات جغرافیایی (Geographic information system یا GIS) و روش‌های تصمیم‌گیری چند معیاره بهره می‌برد. روش‌ها: در این تحقیق از فرایند وزن‌دهی سلسله مراتبی معکوس (Inversion Hierarchical Weighing Process یا IHWP) و نرم‌افزار ArcGIS برای مکان‌یابی محل بهینه دفن پسماند استفاده گردید. بدین منظور، 12 لایه اطلاعاتی اکولوژیکی و افتصادی- اجتماعی طبق روش IHWP وزن‌دهی شد و نقشه مطلوبیت منطقه برای دفن پسماند با استفاده از توابع همپوشانی نقشه‌ها و نیز به کمک 9 لایه محدودیت به‌ دست آمد. یافته‌ها: با توجه به یافته‌های حاصل از طبقه‌بندی نقشه نهایی، 5/91 درصد از سطح منطقه مورد مطالعه از وضعیت کاملاً نامطلوبی برای دفن پسماند برخوردار بود. محدوده‌های با مطلوبیت بسیار زیاد، تنها 16/0 درصد از مساحت شهرستان را به خود اختصاص داد که در نهایت از بین این مناطق، محدوده‌ای به وسعت 44 هکتار در فاصله تقریبی 4 کیلومتری شمال غربی شهر سنندج به عنوان اولویت اول برای دفن پسماند شناسایی و معرفی گردید. نتیجه‌گیری: روش IHWP می‌تواند به عنوان روش نوینی جهت تلفیق لایه‌های اطلاعاتی طبیعی و مصنوعی در پروژه‌های با اهداف مکان‌یابی از جمله مکان‌یابی محل دفن زباله‌های شهری به کار رود و در صورتی که با بررسی‌های میدانی همراه شود، می‌تواند بهترین گزینه‌های تصمیم‌گیری در رابطه با هدف را ارایه نماید.

Title: بررسی ارتباط بین نمره تعارض خانوادگی از دیدگاه دختران دبیرستانی و مادران آن‌ها

http://hsr.mui.ac.ir/index.php/jhsr/article/view/3022

مقدمه: شکل‌گیری تعارضات بین والدین و نوجوانان همواره از مشکلات رایج دوره نوجوانی است که در این دوره رخ می‌دهد. با توجه به این امر، مطالعه حاضر به منظور بررسی ارتباط بین نمره تعارض خانوادگی از دیدگاه دختران دبیرستانی و مادران آن‌ها در شهر اصفهان انجام گردید. روش‌ها: مطالعه از نوع توصیفی- تحلیلی بود. اطلاعات با استفاده از پرسش‌نامه تعارضات خانوادگی از دیدگاه نوجوانان و والدین جمع‌آوری شد. جامعه پژوهش را 220 نفر از مادران و دانش‌آموزان دختر دبیرستانی ناحیه 3 شهر اصفهان تشکیل دادند. پس از تکمیل پرسش‌نامه‌ها توسط گروه هدف، داده‌ها وارد نرم‌افزار SPSS گردید و توسط آزمون‌های همبستگی Spearman’ آزمون t مستقل و آنالیز واریانس یک طرفه مورد تجزیه و تحلیل قرار گرفت. یافته‌ها: بیشترین میزان تعارض بین دانش‌آموزان و مادران در حیطه‌های بلوغ جنسی، وضعیت تحصیلی و نوع ارتباط والدین با یکدیگر و کمترین میزان تعارض در حیطه‌های استقلال‌طلبی و آزادی نوجوان وجود داشت. نتیجه‌گیری: لازم است که در جهت کاهش تعارض بین مادران و نوجوانان در حیطه‌های بلوغ جنسی، وضعیت تحصیلی و نوع ارتباط والدین با یکدیگر مداخله آموزشی مناسب طراحی و ارایه گردد.

Title: پیش‌بینی کننده‌‌های اضطراب بارداری و زیرمقیاس‌های آن در زنان باردار شهر بیرجند در سال 1395

http://hsr.mui.ac.ir/index.php/jhsr/article/view/3172

مقدمه: دوران بارداری به عنوان یکی از مهم‌ترین دوره‌های زندگی زنان، با تغییرات فیزیکی، روانی و اجتماعی خاصی همراه است، اما این تغییرات همیشه مطلوب نبوده و گاهی باعث نگرانی و اضطراب مادر می‌شود. مطالعه حاضر با هدف تعیین پیش‌بینی کننده‌های اضطراب بارداری و زیرمقیاس‌های آن در زنان باردار شهر بیرجند انجام شد. روش‌ها: مطالعه جاری توصیفی- تحلیلی در سال 1395 بر روی 380 مادر باردار مراجعه کننده به مراکز بهداشتی درمانی شهر بیرجند انجام گرفت. نمونه‌گیری به روش چندمرحله‌ای تصادفی انجام شد. از پرسش‌نامه اطلاعات فردی و سوابق بارداری، پرسش‌نامه اضطراب بارداری Vandenberg و پرسش‌نامه رضایت زناشویی MSQ (Marital satisfaction questionnaire) برای جمع‌آوری داده‌ها استفاده شد. داده‌ها در نرم‌افزار SPSS با استفاده از آزمون‌های آنالیز کوواریانس یک متغیره و چند متغیره تحلیل گردید. یافته‌ها: میانگین نمره اضطراب بارداری 13/1 ± 78/3 (دامنه تغییرات 7-1) بود و نشان داد که 54 درصد از مادران اضطراب را در بارداری تجربه می‌کردند. متغیرهای تعداد فرزند، رضایت زناشویی، جنسیت جنین، خواسته بودن جنس جنین در مجموع 7/17 درصد از تغییرات اضطراب بارداری را پیش‌بینی کرده بودند. متغیرهای سن مادر، بارداری خواسته، تحصیلات مادر و همسر، وضعیت اشتغال مادر و همسر، هفته بارداری و درآمد خانوار پیش‌بینی کننده اضطراب بارداری کل نبود. نتیجه‌گیری: با توجه به شیوع بالای اضطراب بارداری، پیشنهاد می‌شود که کارشناسان سلامت با در نظر گرفتن متغیرهای پیش‌بینی فوق، مادران باردار پرخطر را از نظر وضعیت روانی مورد غربالگری قرار دهند تا در صورت لزوم تحت مداخلات آموزشی و روان‌شناختی قرار گیرند.

Title: بررسی رفتارهای خودمراقبتی و ارتباط آن با سواد سلامت در بیماران مبتلا به پرفشاری خون شهرستان اصفهان در سال 95-1394

http://hsr.mui.ac.ir/index.php/jhsr/article/view/2977

مقدمه: با توجه به این که رعایت رفتارهای خودمراقبتی در بیماران مبتلا به پرفشاری خون از عوامل اصلی کنترل پرفشاری خون می‌باشد و نقش سواد سلامت در کنترل این بیماری حایز اهمیت است، از این‌رو، مطالعه حاضر با هدف تعیین رفتارهای خودمراقبتی و ارتباط آن با سواد سلامت در بیماران مبتلا به پرفشاری خون شهرستان اصفهان انجام گرفت. روش‌ها: پژوهش حاضر یک مطالعه مقطعی بود که بر روی 210 بیمار مبتلا به پرفشاری خون مراجعه کننده به مراکز سلامت منتخب شهرستان اصفهان انجام گرفت. روش نمونه‌گیری، تصادفی چندمرحله‌ای بود و از پرسش‌نامه‌های خودمراقبتی در فشار خون بالا HSCALE (Self- care in hypertension patient) و فرم کوتاه پرسش‌نامه سواد سلامت عملکردی بزرگسالان STOFHLA (Functional health literacy in adult) استفاده شد. اطلاعات جمع‌آوری شده با استفاده از آزمون‌های t مستقل، ضریب همبستگی Pearson، همبستگی Spearman و رگرسیون خطی چندگانه در نرم‌افزار SPSS مورد تجزیه و تحلیل قرار گرفت. یافته‌ها: میانگین سنی افراد مورد مطالعه 9/8 ± 3/57 سال بود. میانگین نمره رفتارهای خودمراقبتی و نمره سواد سلامت در شرکت کنندگان به ترتیب 01/12 ± 02/150 از 190 و میانگین نمره سواد سلامت آن‌ها 73/14 ± 83/75 از100 به دست آمد. درصد پایینی از بیماران (1/7 درصد) رژیم کم نمک داشتند و 39 درصد آنان فشار خون خود را مرتب اندازه‌گیری می‌کردند. 3/74 درصد بیماران سواد سلامت کافی داشتند، اما ارتباط آماری معنی‌داری بین سواد سلامت و رفتارهای خودمراقبتی یافت نشد (631/0 = P). نتیجه‌گیری: رفتارهای خودمراقبتی بیماران در حد مطلوبی بود و درصد بالایی از افراد مورد مطالعه سواد سلامت کافی داشتند، اما رفتارهای خودمراقبتی با سواد سلامت ارتباط نداشت. بنابراین، لازم است تا آموزش بیماران متناسب با سطح سواد سلامت و رفتارهای خودمراقبتی آنان در مراکز سلامت صورت گیرد.

Title: امکان‌سنجی پایش بیولوژیک آهن سه ظرفیتی و روی در ادرار با روش ریزاستخراج بر پایه‎ قطره آلی جامد شناورپایش بیولوژیک آهن سه ظرفیتی و روی در نمونه های ادرار با روش ریز استخراج بر پایه قطره آلی جامد شناور

http://hsr.mui.ac.ir/index.php/jhsr/article/view/3018

مقدمه: اهمیت نقش شیمی تجزیه در بهداشت حرفه‌ای و مباحث سم‌شناسی، بسیار برجسته و حیاتی می‌باشد. به این منظور، برخی روش‌های استخراج و آنالیز تعریف شده است. در تحقیق حاضر، به منظور استخراج مقادیر بسیار جزیی کاتیون‌های آهن سه ظرفیتی و روی در نمونه‌های ادرار، روش ریزاستخراج بر پایه قطره آلی جامد شناور انتخاب گردید. این روش، به دلیل عدم کاربرد قبلی آن جهت استخراج آهن و روی از نمونه‌های ادرار، مورد بررسی‌ قرار گرفت. روش‌ها: پژوهش حاضر از نوع مطالعات تجربی بوده و جهت استخراج آهن و روی از نمونه‌های ادرار انجام گرفت. تحقیق مذکور در چند مرحله شامل تهیه محلول‌های استاندارد، استخراج به روش ریزاستخراج بر پایه قطره آلی جامد شناور و در نهایت، آنالیز با دستگاه جذب اتمی انجام شد و شرایط بهینه استخراج، راندمان و تکرارپذیری نتایج مورد بررسی قرار گرفت. یافته‌ها: حجم بهینه لیگاند 2 میلی‌لیتر و درصد انحراف معیار نسبی در آنالیز نمونه‌های اسپایک شده آهن و روی در ادرار (08/0 میکروگرم بر میلی‌لیتر) به ترتیب برابر با 6/2 و 4/3 درصد و راندمان برابر با 4/97 و 6/96 درصد بود. همچنین، پارامترهایی مانند حجم حلال و دمای استخراجی تأثیر بسزایی بر راندمان استخراج فلز آهن و روی با روش استخراج مورد نظر داشت. نتیجه‌گیری: روش ریزاستخراج بر پایه قطره آلی جامد شناور، در تعیین مقادیر جزیی آهن و روی در نمونه‌های ادرار موفق بوده وکاهش حجم نمونه و زمان استخراج، کاهش مواجهه اپراتور با مواد سمی، از برجسته‌ترین مزایای این روش می‌باشد.
